# SARS CoV-2 Seroprevalence in Selected States of High and Low Disease Burden in Nigeria

**DOI:** 10.1001/jamanetworkopen.2022.36053

**Published:** 2022-10-11

**Authors:** Olatunji Matthew Kolawole, Oyewale Tomori, Dennis Agbonlahor, Ekanem Ekanem, Rasheed Bakare, Nasidi Abdulsalam, Obehi Okojie, Eka Braide, Benjamin Uzochukwu, Abdulmumini Rafindadi, Shaibu Bello, Sule Shehu, Oye Gureje, Muhammed Lecky, Obinna Onwujekwe, Cajetan Onyedum, Adaobi Ezike, David Bukbuk, Garba Ashir, Bond Anyaehie, Uche Amazigo, Abdulrajak Habib, Joy Ufere, Ngozi Azodoh

**Affiliations:** 1 Ministerial Expert Advisory Committee on COVID-19-Health Sector Response, Federal Ministry of Health, Abuja, Nigeria; 2 Department of Microbiology, Faculty of Science, University of Maiduguri Teaching Hospital, Maiduguri, Borno State, Nigeria; 3 Department of Physiology, College of Medicine, University of Nigeria, Enugu; 4 Pan-African Community Initiative on Education and Health, Enugu, Enugu State, Nigeria; 5 World Health Organization Country Office, Federal Capital Territory, Abuja, Nigeria

## Abstract

**Importance:**

The global impact of COVID-19 has led to an increased need to continuously assess disease surveillance tools. The utility of SARS-CoV-2 serologic tools in determining immunity levels across different age groups and locations in helping to quickly assess the burden of COVID-19 with significant health policy implications is unknown.

**Objective:**

To determine the prevalence of SARS-CoV-2 antibodies with respect to the age group and sex of participants.

**Design, Setting, and Participants:**

A cross-sectional survey of 4904 individuals across 12 states with high and low COVID-19 disease burden in Nigeria was carried out between June 29 and August 21, 2021.

**Main Outcomes and Measures:**

Enzyme-linked immunosorbent assay was used for the detection of specific SARS-CoV-2 immunoglobulin G and immunoglobulin M antibodies, such as the nucleocapsid protein-NCP and spike protein S1. Interviewer-administered questionnaires provided information on participants’ history of disease and associated risk factors.

**Results:**

A total of 4904 individuals participated in the study (3033 were female [61.8%]; mean [SD] age, 26.7 [6.51] years). A high seroprevalence of SARS-CoV-2 (78.9%) was obtained. Seropositivity was consistent across the states surveyed, ranging from 69.8% in Lagos to 87.7% in Borno. There was no association between sex and seropositivity (female, 2414 [79.6%]; male, 1456 [77.8%]; *P* = .61); however, an association was noted between age and seropositivity, with the peak prevalence observed in participants aged 15 to 19 years (616 [83.6%]; *P* = .001). Similarly, loss of appetite (751 [82.3%]; *P* = .04) and smell (309 [84.4%]; *P* = .01) were associated with seropositivity.

**Conclusions and Relevance:**

In this cross-sectional study, a high SARS-CoV-2 seroprevalence was obtained among the study population during the low level of vaccination at the time of the survey. Thus, there is a need for both an efficacy and antibody neutralization test study to ascertain the efficacy of the antibody detected and the potential for herd immunity in Nigeria.

## Key Points

**Question:**

What is the serologic prevalence of SARS CoV-2 in states with high and low disease burdens in Nigeria?

**Findings:**

In this cross-sectional study including 4904 participants, a high seroprevalence of 78.9% was obtained across 12 states in Nigeria. Seropositivity was consistent across the states surveyed, ranging from 69.8% in Lagos to 87.7% in Borno.

**Meaning:**

The results from this study suggest that COVID-19 infection is prevalent in Nigeria despite the low hospitalization rate recorded at the time of sampling.

## Teaser

This cross-sectional study examines the seroprevalence of SARS-CoV-2 in 12 states with high and low disease burden throughout Nigeria from June 29 to August 21, 2021.

## Introduction

COVID-19 has dominated virtually every aspect of human existence in the past 3 years. This disease has had enormous influence on mortality worldwide. The economic impact of COVID-19 has been massive, with almost all the countries of the world undergoing varying degrees of lockdowns in the past 3 years. As of September 10, 2022, Nigeria had recorded 264 014 confirmed cases, with 257 510 patients discharged from the hospital and 3356 deaths recorded in 36 states and the Federal Capital Territory.^1^

The global impact of COVID-19 has led to an increased need to continuously assess disease surveillance tools. Disease surveillance is a vital tool for the assessment of the burden or extent of health problems that informs the identification of high-risk groups, infection trends, and areas of a target for appropriate interventions. Surveillance via epidemiologic and laboratory trends is usually used in routine monitoring of disease. However, the use of serologic evidence is most suitable in the determination of immunity levels and the presence of a particular disease across different age groups and locations.^2^

Assessing the burden of COVID-19 infection based on medically attended cases comprising only individuals with disease manifestation or complications resulting from SARS-CoV-2 infection is inadequate. This limitation is largely due to the gaps in the dynamics of case definition, testing strategies, and the high proportion of asymptomatic individuals in the population. Consequently, serosurveys for the detection of SARS-CoV-2 antibodies in the general population may provide a more credible and accurate estimate of the burden of infection and progression of the epidemic.

The seroprevalence of SARS-CoV-2 infection, based on the detection of virus-specific antibodies IgG and IgM among various populations in China, the US, Switzerland, Spain, Italy, and some other European and South American countries, as well as in some African countries (Kenya, Tanzania, and Zambia) has been documented. These rates have varied across countries and cities: China, 3.2% to 3.8%^3^; Los Angeles, 4.06%^4^; Spain, 5.0%^5^; Switzerland, 4.8%^6^; Kenya, 5.6% to 9.5%^7^; and Malawi, 12.3%.^8^

In Nigeria, a few seroprevalence studies have been carried out at different times,^9,10^ with the prevalence recorded in these studies influenced by the stage of the epidemic in the area/location of the study. The prevalence recorded in these studies is largely influenced by the number of days between the first report of COVID-19 in the locations and the sampling time. Thus, this study was carried out to determine the seroprevalence of SARS-CoV-2 IgG and IgM antibodies with respect to age group and sex of participants, estimate the fraction of the populace with asymptomatic or subclinical COVID-19 infections, determine and confirm the associated signs and symptoms of COVID-19 infection with the serostatus of participants, and provide an enhanced understanding of the antibody dynamics of COVID-19 infection.

## Methods

### Study Design and Location

This was a 1-time cross-sectional investigation across selected states with high and low disease burden in the 6 geographic zones in Nigeria. To estimate the overall burden of the circulation of SARS-CoV-2 infection in Nigeria, strategic locations/geographic areas based on data from the Nigerian Centre for Disease Control were used.^1^

Ethical and institutional approval was obtained from the National Health Research Ethics Committee and respective health facilities across the selected states before the commencement of the study. Informed consent was obtained from the participants or their guardians as appropriate. Each individual was informed that participation in the investigation was voluntary and that they were free to withdraw, without the need for justification, from the investigation at any time without consequences and without affecting professional responsibilities. Participants did not receive financial compensation. This study follows the Strengthening the Reporting of Observational Studies in Epidemiology (STROBE) reporting guideline for cross-sectional studies.

Twelve states were chosen based on COVID-19 prevalence at the time of sampling. The areas were across locations with high and low incidences of infection in each of the 6 geopolitical zones of Nigeria, including Abuja and Kwara (North Central), Kaduna and Sokoto (Northwest), Edo and Akwa-Ibom (South-South), Lagos and Ondo (Southwest), Enugu and Imo (Southeast), and Borno and Yobe (Northeast) (Figure).

**Figure.**
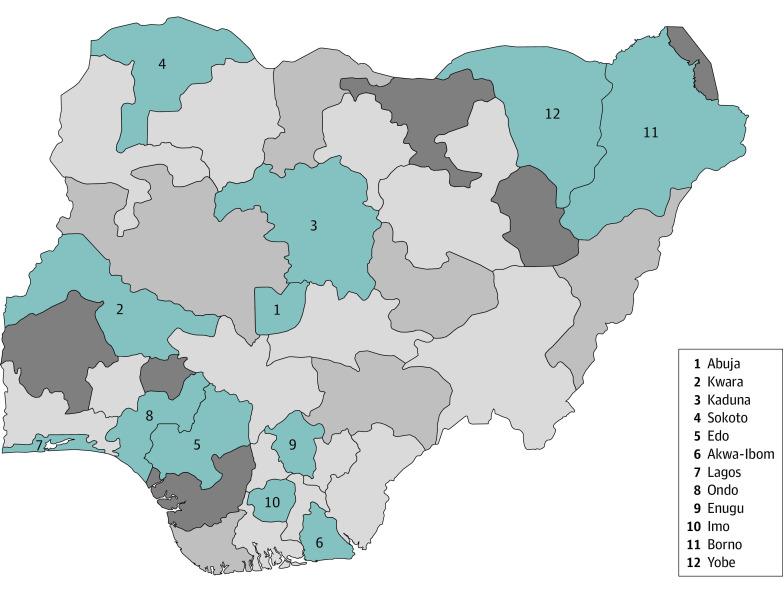
Incidence of Infection Throughout Surveyed Locations in Nigeria

### Recruitment and Data Collection

The selection of government-owned hospitals and school clinics was restricted to state capitals. Participants were recruited via convenient random sampling of individuals visiting general hospitals in communities and school clinics to successfully capture data across different age groups, occupations, and sexes. The sampling period was from June 29 to August 21, 2021, during the second wave of the pandemic. At the time of the survey, the AstraZeneca vaccine was the only vaccine being used in the country and it was not widely circulated.

All individuals, irrespective of age, sex, symptoms of infection, or history of COVID-19 infection, were included. There were no exclusion criteria. Using a structured questionnaire administered online, data of demographic and clinical information relating to the participants and COVID-19 transmission and symptoms were collected to evaluate the epidemiologic parameters.

### Serologic Testing

Blood samples were collected and centrifuged to obtain serum that was used for serologic analysis. Enzyme-linked immunosorbent assay (BeijingWantai BioPharm) was used for the detection of specific SARS-CoV-2 IgG and IgM antibodies, such as the nucleocapsid protein-NCP, spike protein S1, and assay for IgG and IgM. The anti-SARS-CoV-2/anti-SARS-CoV-2 NCP serologic kit approved by the US Food and Drug Administration SARS-CoV-2 Ab ELISA (BeijingWantai BioPharm) for antibody detection was procured by the World Health Organization as part of the unity seroprevalence study protocol in Africa. The serology kit has a sensitivity of 96.7% and specificity of 97.5%.

### Statistical Analysis

The collected data were analyzed using the χ^2^ test. Correlations were determined using Pearson analysis, and significance was indicated at a 2-sided paired value of *P* < .05. Analysis was done using Microsoft Excel (Microsoft Corp) and SPSS, version 20 (SPSS Institute LLC).

## Results

A total of 4904 individuals (3033 female [61.8%], male 1871 [38.2%]; mean [SD] age, 27.6 [6.51] years) participated in the study. The age group 20 to 29 years was the most represented (1004 [20.5%]) in the study and the age group 70 years and older (80 [1.6%]) was the least represented (Table 1). The overall seroprevalence of SARS-CoV-2 was 78.9%. Of the 4904 tested samples, 3870 were positive (78.9%) and 1034 were negative (21.1%) (Table 2). Seropositivity was consistent across the states, ranging from 69.8% in Lagos to 87.7% in Borno (Table 2). There was, however, no statistically significant difference noted between state/geographic zones and seropositivity.

**Table 1.** Age and Sex Distribution of the Participants

Demographic characteristic	Total, No. (%)
No.	4904
Age group, y	
0-4	318 (6.5)
5-9	323 (6.6)
10-14	596 (12.2)
15-19	737 (15.0)
20-29	1004 (20.5)
30-39	884 (18.0)
40-49	546 (11.1)
50-59	275 (5.6)
60-69	141 (2.9)
≥70	80 (1.6)
Sex	
Female	3033 (61.8)
Male	1871 (38.2)

**Table 2.** SARS-CoV-2 Seropositivity per State Surveyed in Nigeria

State	No. (%)		Total
	Negative	Positive	
Abuja	97 (24.2)	304 (75.8)	401
Akwa-Ibom	84 (18.9)	360 (81.1)	444
Borno	48 (12.3)	341 (87.7)	389
Edo	108 (26.0)	308 (74.0)	416
Enugu	58 (14.1)	354 (85.9)	412
Imo	77 (19.3)	323 (80.8)	400
Kaduna	95 (23.2)	315 (76.8)	410
Kwara	95 (23.8)	305 (76.3)	400
Lagos	121 (30.2)	280 (69.8)	401
Ondo	111 (26.4)	310 (73.6)	421
Sokoto	60 (15.0)	340 (85.0)	400
Yobe	80 (19.5)	330 (80.5)	410
Total	1034 (21.1)	3870 (78.9)	4904

In this study, there was no significant difference in the SARS-CoV-2 positivity rates among women (2414 [79.6%]) and men (1456 [77.8%]; *P* = .61). Participants aged 10 to 44 years recorded the highest COVID-19 seropositivity; those aged 0 to 4 years had the lowest level. There was an association between the age of the participants and seropositivity (χ^2^ = 146.59; *P* < .001).

With respect to COVID-19 vaccination, 254 of the total 4904 participants (5.2%) stated they had received the first and second doses of the Oxford-AstraZeneca COVID-19 vaccine. Participants aged 20 to 59 years had the highest rate of reported vaccination (Table 3), and more women (n = 143) were vaccinated than men (n = 111). An association was found between COVID-19 vaccine uptake and seropositivity, despite weighting for respondents with unknown COVID-19 vaccine status (616 [83.6%]; *P* = .001). Likewise, an association was noted between the age distribution of the participants and their COVID-19 vaccination status (616 [83.58%]; *P* = .001). In accessing the risk factors associated with COVID-19, only loss of smell (309 [84.4%]; *P* = .01) and loss of appetite (751 [82.3%]; *P* = .04) showed a statistical correlation with SARS-CoV-2 seropositivity.

**Table 3.** Association Between Age of the Participants and SARS-CoV-2 Seropositivity^a^

Age range, y	Seropositive	Seronegative	Total
0-4	186 (58.49)	132 (41.51)	318
5-9	216 (66.87)	107 (33.13)	323
10-14	450 (75.5)	146 (24.5)	596
15-19	616 (83.58)	121 (16.42)	737
20-24	422 (84.74)	76 (15.26)	498
25-29	414 (81.82)	92 (18.18)	506
30-34	393 (82.22)	85 (17.78)	478
35-39	335 (82.51)	71 (17.49)	406
40-44	252 (81.55)	57 (18.45)	309
45-49	191 (80.59)	46 (19.41)	237
50-54	140 (84.85)	25 (15.15)	165
55-59	90 (81.08)	21 (18.92)	111
60-64	67 (75.28)	22 (24.72)	89
65-69	40 (78.43)	11 (21.57)	51
≥70	58 (72.5)	22 (27.5)	80
Total	3870 (78.92)	1034 (21.08)	4904

^a^ Findings significant at χ^2^ = 146.59; *P* < .001.

## Discussion

In this study, a total of 4904 individuals across 12 states in Nigeria were surveyed, making it one of the most widespread COVID-19 serosurveys conducted in Nigeria to date. The use of disease surveillance experts as well as integrating an online process for submission and analysis of questionnaires collated from the field improved the implementation of this study. In addition, the study was carried out across the 6 different geographic zones in Nigeria to ensure that confounding variables associated with culture and lifestyles were accounted for.

The seroprevalence of SARS-CoV-2 in this study was 78.9%. This is the highest seroprevalence of SARS-CoV-2 that has been recorded in Nigeria and one of the highest recorded in Africa. The high seroprevalence of SARS-CoV-2 obtained can be attributed to the type of assay used. The kits used at that time contained assays for both IgG and IgM, which potentially increases positivity rates compared with kits that assay for either IgG or IgM. In addition, the high prevalence obtained in Nigeria can be attributed to the sampling period, which occurred at the onset of the second wave of the pandemic in Nigeria, as there is a connection between sampling period and SARS-CoV-2 seropositivity. Different studies conducted in Nigeria between June and December 2020 showed varying seroprevalence findings ranging from 17% to 25.41%.^9,10^ The association between period of sampling and seropositivity is further seen in a meta-analysis of SARS-CoV-2 seroprevalence in Africa across 23 studies and 27 735 individuals, with a pooled seroprevalence of 22%.^11^ Most reports in Africa have shown a distinctively low seroprevalence in different regions.^12,13,14^ Despite this finding, the study in South Africa^15^ had a high seroprevalence of 63%, which is similar to the high prevalence obtained in this study. A distinct feature from this study in South Africa^15^ and the other 22 studies in the meta-analysis is the sample collection time. Only the study by Sykes et al^15^ and our study had samples collected in 2021. A major moderator of the prevalence COVID-19 is the number of days between the time of the first reports in these locations and the time of the studies.^11^ Thus, the period between the first onset of COVID-19 in Nigeria and the time of sampling could account for the high seropositivity obtained in this study. Furthermore, the higher SARS-CoV-2 antibody prevalence in Nigeria as reported in this study compared with previous studies reflects on both the interval between the first report of COVID-19 cases and the time of the study and the number of COVID-19 waves reported in the country.^16^ This association was also reported in other countries, such as Ireland, where the increase in seroprevalence reflects the magnitude of the third wave of the pandemic.^17,18^

The high seroprevalence of SARS-CoV-2 in Nigeria as seen in this study could be attributed to several factors, including the possibility of other circulating variants of SARS-CoV-2 in Nigeria. Nigeria has recorded different cases of the Delta variant and recent cases of the Omicron variant. With the Delta variant already shown to be more contagious and resulting in increased COVID-19 transmissibility, there is a likelihood of this characteristic contributing to the high prevalence of COVID-19 as recorded in this study. A nexus can be drawn between the high seroprevalence of SARS-CoV-2 in Nigeria and South Africa^15^ and the presence of these multiple variants of COVID-19 in both countries.

Other factors that could be involved in the high seroprevalence of SARS-CoV-2 as seen in this study include the lack of strict adherence to prevention and control measures, such as social distancing, use of masks, and handwashing habits. In some instances, people believe that COVID-19 is a hoax.^19^ Adherence to these measures in a low-income and-medium-income country, such as Nigeria, is somewhat challenging because various factors, including financial need, lack of personal hygiene, ignorance, mistrust of leaders, and lack of political will, make strict enforcement of these protective measures difficult.^19^

Results from this study showed a consistent seroprevalence across all the states surveyed despite the diverse sociocultural attributes of the participants across the geographic zones, suggesting that sociocultural distribution did not influence the prevalence of COVID-19. There was no association between states or geographic zones and seropositivity. This finding is in tandem with reports^20,21,22^ that have also noted no correlation between geographic zones and SARS-CoV-2 seroprevalence. The consistent interstate travels for business, education, leisure, and tourism are factors that could increase the propagation of COVID-19 infection. In this study, major cities were primarily surveyed, and these areas attract the inflow and outflow of individuals for various reasons across the country. This traffic, coupled with the lack of adherence to prevention measures, allows for the easy transmission of SARS-CoV-2 and exposure to infection across different states in Nigeria.

The findings from this study showed no association between sex and seropositivity. This is in line with various global data pools that reported that sex does not significantly influence SARS-CoV-2 seropositivity and transmissibility.^23,24,25^ The World Health Organization and African Centre for Disease Control have also indicated that sex has no role in SARS-CoV-2 seropositivity.^26^ There was, however, an association between the age group of the participants and seropositivity. The highest level of seropositivity was reported among the age group 10 to 44 years, with a peak prevalence observed among the age group 15 to 19 years. This finding is similar to global reports that show a high prevalence of COVID-19 among the population.^27,28,29,30^

In this cross-sectional study, COVID-19 vaccine uptake was found to be associated with seropositivity after weighting for participants who did not know their COVID-19 vaccine status. This finding suggests that there is a need for a vaccine efficacy study to have a clearer understanding of the association between seropositivity and vaccination. Sauré et al^31^ made similar observations, reporting that seropositivity increased over time after increased vaccine uptake. Nevertheless, as emphasized in the reports of DeFrancesco^32^ and Lechien,^33^ there is a need for further studies on immune responses as well as vaccine efficacy and outcomes to fully understand the association of vaccine uptake with SARS-CoV-2 seropositivity.

A major finding in our study is the association between the loss of smell and loss of appetite among the participants and SARS-CoV-2 seropositivity. A high level of seropositivity was noted in persons with a history of loss of smell and appetite. A similar report was made by Mercante et al,^34^ who reported that a high prevalence of anosmia and ageusia was common among SARS-CoV-2–positive patients. Other studies have also observed loss of smell and loss of appetite as factors associated with COVID-19.^34,35^

### Limitations

This study has limitations. A major limitation is that sampling across the 12 states surveyed was carried out in hospitals and school clinics in city centers. Thus, the results should be interpreted with caution. Similar studies randomly sampling inhabitants of rural communities may present different results.

## Conclusions

Overall, a high seroprevalence of SARS-CoV-2 was found in this study. This was consistent across the different states in Nigeria surveyed, despite the diverse sociocultural attributes of the participants across these states. The presence of multiple variants of COVID-19 (Delta and Omicron) with improved ability to increase the transmissibility of the disease in Nigeria could be responsible for the high level of seropositivity obtained. Thus, there is a need for both an efficacy and antibody neutralization test study to ascertain the efficacy of the antibody detected and the potential for herd immunity in Nigeria.
